# Longevity of the Adult Codling Moth, *Cydia pomonella,* and the Obliquebanded Leafroller, *Choristoneura rosaceana,* in Washington Apple Orchards

**DOI:** 10.1673/031.008.1401

**Published:** 2008-02-22

**Authors:** Vincent P. Jones, Nik G. Wiman

**Affiliations:** Department of Entomology, Tree Fruit Research and Extension Center, Washington State University, 1100 N. Western Ave., Wenatchee, WA 98801

**Keywords:** codling moth, obliquebanded leafroller, field longevity, phenology

## Abstract

The longevity of adult codling moth (*Cydia pomonella* (L.) Lepidoptera: Tortricidae) and obliquebanded leafroller (*Choristoneura rosaceana* (Harris) Lepidoptera: Tortricidae) held in shaded vials in the tree canopy was measured during the normal flight periods during 2004 and 2005. In both years all codling moths were dead by 130 degree-days (DD) (21 d) in the spring and 121 DD (8 d) in the summer. On a degree-day basis, data were similar across sex, generation, and year, and a common curve adequately predicted the survival distribution. For the obliquebanded leafroller, there were longevity differences between the sexes, but not between generations or years. Use of empirical quantile-quantile plots showed that the female obliquebanded leafroller lived an average of 32% longer than males. Maximum longevity observed in these studies for obliquebanded leafrollers was 117 DD (11 d) across both generations. The implications of these data for population biology studies and quarantine requirements are discussed.

## Introduction

The codling moth, *Cydia pomonella* (L.) (Lepidoptera: Tortricidae), is considered one of the most important pests of apples worldwide (Geier 1963; Barnes 1991; Sæthre and Hofsvang 2002; Evenden and McClaughlin 2005; Kührt et al. 2006), and the obliquebanded leafroller, *Choristoneura rosaceana* (Harris) (Lepidoptera: Tortricidae) is a severe problem in most apple producing areas in North America (Beers et al. 1993; Waldstein and Reissig 2001; Fadamiro 2004; Trimble and Appleby 2004; Wilkinson et al. 2004; Evenden and McClaughlin 2005). Despite the importance of these pests to apple production, there are still aspects of their population biology where information is missing or poorly quantified.

Knowledge gaps have become more apparent as apple pest management moves away from strict pesticide-based management programs and towards biological control and behaviorally based systems such as mating disruption (Jones et al. 2005a). Of particular importance is the need to understand their basic population biology that may help explain the mechanisms of mating disruption that act to reduce population growth (Barclay and Judd 1995), such as delay of mating (Jones and Aihara-Sasaki 2001). Laboratory studies are crucial to the effort to understand population dynamics, but the studies must be related back to the field if there is any hope of understanding population growth. Life tables are frequently used as a tool in laboratory studies to help understand population growth, primarily because age-specific mortality and reproductive rates can be quantified in a way that allows simple population models to be developed (Carey 1993). However, a major problem with this approach is that lab studies are typically run under nearly ideal conditions of temperature and humidity, rather than the variable conditions encountered in the field. Lab studies are particularly useful in generating age-specific reproductive rates, which are nearly impossible to develop in most field situations. Unfortunately, longevity estimates based on lab studies are typically extremely optimistic, and can affect the perception of population biology. For example, if moth longevity in the field is much shorter than lab studies indicate, then the effects of delaying mating by even a few days would greatly restrict population growth making mating disruption more effective. This would be true even if the delay in mating did not result in a reduction of the number of fertile eggs laid per day or over the life span of the female, simply because the oviposition period would be shorter. The problems with laboratory longevity studies are especially true in studies that provide alternate food sources (e.g., honey water mixtures or the like) that would be rare or non-existent in the environment of interest.

It is important to make the distinction between an age-specific survivorship curve (*l_x_* curve) and survival estimates based on simple summary statistics (e.g., mean or maximum survival time). Age-specific survival curves provide a comprehensive, quantitative estimate of mortality experienced by the population over time (Carey 1993), whereas summary statistics attempt to define a single statistic that describes the “typical” mortality experienced. Commonly used summary statistics, such as the mean, can lead to questionable conclusions, as they may be highly influenced by extreme values (Mosteller and Tukey 1977). Attempts to derive a survivorship curve using means and standard deviations (or SEM) by assuming the normal distribution are flawed because there are many possible survivorship curve shapes (Carey 1993). With *C*. *pomonella* in particular, these simple summary statistics are virtually all that are available (Geier 1963; Hagley 1972; Howell 1981), and this restricts our understanding of population processes occurring in the field. With *C*. *rosaceana*, longevity estimates are even more limited (Sanderson and Jackson 1909; VPJ and NGW unpublished observations). Finally, having the *l_x_* curve on a physiological time (degree-day) basis (Taylor 1981) allows the link between models with field conditions to help explain population dynamics at various times during the season.

Another key use of longevity data is to provide a biological basis for quarantine regulations. In particular, quarantines are typically designed to prevent establishment or maintain a low risk of establishment of a new pest insect (Hallman 2002). Thus, multiple pest introductions in commodities (such as *C*. *pomonella* in apple shipments) may trigger a complete shut down of a market, regardless of the time between introductions within a season (APHIS-PPQ 2005). The ability to reasonably predict survival rates would allow estimation of intervals between accidental introductions that would insure minimal risk of establishment and spread of pests.

**Table 1.**
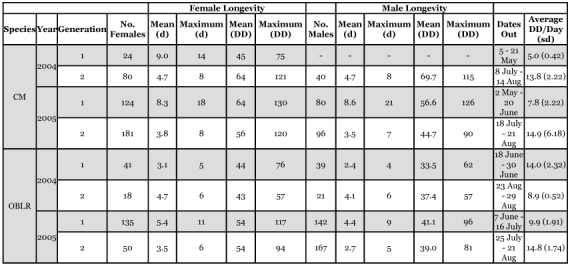
Numbers of CM and OBLR used to determine longevity in apple orchards at different times in the season and longevity in calendar days and degree-days (DD).

These studies were implemented to determine the longevity of *C*. *pomonella* and *C*. *rosaceana* under field conditions in Washington apple orchards. We examined longevity of both sexes on a degree-day (heat unit or physiological time) basis to determine if longevity could be predicted throughout the flight period of both species.

## Materials and Methods

*C*. *pomonella* used in the experiments were reared on an artificial diet and obtained from the USDA-ARS YARL lab (Wapato, WA) and were the non-diapausing strain. Moths were shipped to Washington State University's Tree Fruit Research and Extension Center (TFREC) in Wenatchee, WA as last instar larvae and pupae in cardboard bands. In the laboratory, the pupae were removed from the bands, sorted by sex, and placed in cages for emergence in a temperature cabinet (22°C, RH ≈ 75 %) with a photoperiod synchronized each week with the naturally occurring sunrise and sunset. Cages were examined daily for emergence and newly emerged individuals were placed in a vial (160 cm^3^) with screen on both ends to allow free air movement. These vials were then placed within large plastic delta traps (Suterra LLC, Bend, OR 97702) that were hung inside an apple tree canopy at the Tree Fruit Research Center during the normal time of adult flight in both generations per year. The numbers of *C*. *pomonella* placed in the orchard at any given time were variable, depending on colony production (Table 1). All moths used in the experiments (of both sexes) were virgins and were never allowed to mate. The date each moth was placed in the orchard, its sex, and the day it died were recorded.

*C*. *rosaceana* moths were obtained from the colony maintained by the Tree Fruit Research Center, which was reared on an artificial pinto bean diet (Shorey and Hale 1965). Last instar caterpillars were placed in plastic cups (96 cm^3^) and allowed to pupate. After pupation, the same handling and processing methods described for codling moth were applied.

The use of the delta trap/vial combination was viewed as a reasonable compromise that eliminated predation (which would be an orchard-specific mortality factor), and by hanging the trap/vial combination within the tree canopy, would reasonably approximate the ability of the moths to move to cooler locations within the tree so that heat stress would be reduced. Moths would have had access to any dew that formed on the inside of the vials, but no food or water was provided because honeydew from aphids, scales, or mealybugs is virtually non-existent in commercial apple orchards. As such, the estimates of longevity are still likely to be an overestimate of moth longevity compared to actual longevity in the orchard where predators and pesticide residues would be encountered. In addition, confining the moths likely increased longevity because the energy demands normally associated with flight are greatly reduced. Regardless, the longevity estimates reported herein were done under conditions considerably closer to that experienced in nature than typical laboratory derived estimates and additionally, provide a full survivorship curve calculated on a degree-day basis.

The studies were initiated for the spring generation of both species in the spring of 2004 to evaluate the methodology. Samples sizes were increased as much as colony production would allow during the summer generations of 2004 and in both generations during 2005 (Table 1).

## Analysis

Temperature data were collected from the Washington State University AgWeather Net station, located roughly 250 m away from the orchard where the traps were placed. Degree-days (DD) were calculated based on a 10°C base (Riedl et al. 1976; Jorgensen et al. 1979; Pitcairn et al. 1991; Howell and Neven 2000) with no upper threshold using single-sine method (Baskerville and Emin 1969). Although normally both codling moth and *C*. *rosaceana* DD are calculated with an upper threshold of 30°C and 31°C, respectively (Beers et al. 1993; Jones et al. 2005b), the upper threshold was excluded because we wanted to account for high temperature heat effects on longevity. Longevity was calculated for both species in terms of DD lived for each moth.

For each species, longevity was analyzed separately for each sex. Analysis was performed by taking all the individuals over a particular generation, determining their age in DD at death, and constructing an *l_x_* curve. Briefly, an *l_x_* curve is the age-specific survival of individuals expressed as a proportion of the original cohort alive at each period. In all situations, Gompertz distributions were fit to the *l_x_* curves (Carey 1993) using a non-linear routine in Stata (Statacorp 2005). The Gompertz distribution is defined as:

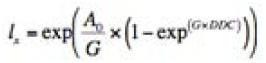
where *A_0_* is the initial mortality rate, *G* is the rate of senescence, and DDC is the time in degree-days since adult emergence.

Because the *l^x^* curves were based on different sample sizes between generations, the fit was weighted using the inverse of the binomial variance for each point (i.e.,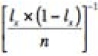) where *l_x_* is the proportion survival at a particular point in time and *n* is the number of individuals that were used to develop the particular *l_x_* curve.

Empirical quantile-quantile (*q-q*) plots (Chambers et al. 1983) were used to compare survival distributions by sex. Quantile-quantile plots were used because they are simple to calculate and allow quick and visually powerful comparisons between distributions. The plots were developed by sorting the longevity of the groups from smallest to largest and then determining the percentiles of the distribution. The percentiles of the two distributions were then plotted so that the equivalent percentiles (e.g., the first percentile of the data from each distribution, second, etc.) were plotted as a scatter plot. If the two distributions were identical, all the points would lie on a line where *y* = *x.* If the points were on a straight line, but with a slope different than 1, then the distributions are not identical (they are similar in shape, but differ by a constant) (Chambers et al. 1983). The shape may also be different between distributions, suggesting non-linear relationships in percentiles where the distribution shape deviates from *y* = *x.*

## Results

**Figure 1.**
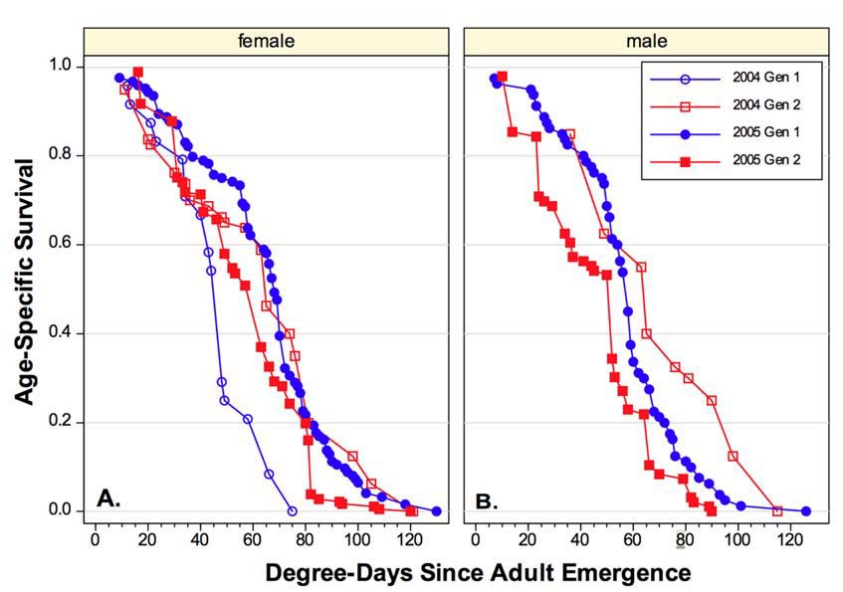
Age-specific survival curves for *Cydia pomonella* on a degree-day basis. A. Females. B. Males.

When the *l_x_* curves for the two years and two generations per year for female *C. pomonella* were plotted on the same axis, the curves for all but generation 1 in 2004 were overlapping across the entire range (Figure 1A). The data for generation 1 in 2004 started at roughly the same rate but dropped rapidly after roughly 40 DD had accumulated. While it is possible that this reflects the variability in mortality between generations or years, it is also possible that it reflects the relatively small sample size (24 females) tested in that year/generation (Table 1). The Gompertz curve fitted to the entire data set was highly significant (*F* = 5127.5, *P* > 0.0001, *df* = 2, 107) and accounted for 99% of the total variation in the data (Table 2). The maximum longevity of *C*. *pomonella* females overall tests was 130 DD or 18 d (Table 1).

The data for male *C. pomonella* was more restricted than the female data, with only one year of data for the spring generation and two years from the summer generation. This data set appeared to be much more homogenous (it lacked the first generation in 2004) with relatively minor differences between the different generations/years (Figure 1B). The Gompertz curve again described the differences well (*F* = 2208.5, *P* > 0.0001, *df* = 2, 72) and accounted for 98% of the total variation in the data (Table 2). Maximum longevity for *C. pomonella* males was 126 DD or 21 d (Table 1)

**Table 2.**
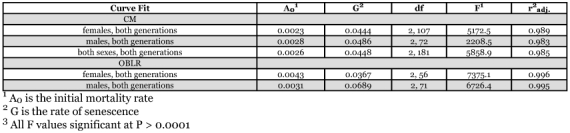
Gompertz fits to codling moth and obliquebanded leafroller survival curves.

**Figure 2.**
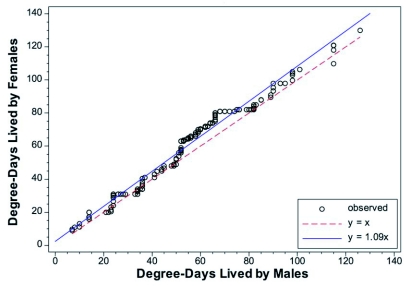
Empirical quantile-quantile plots for male versus female *Cydia pomonella.* If all the data points fell on the *y* = *x* curve, the two distributions would be identical.

**Figure 3.**
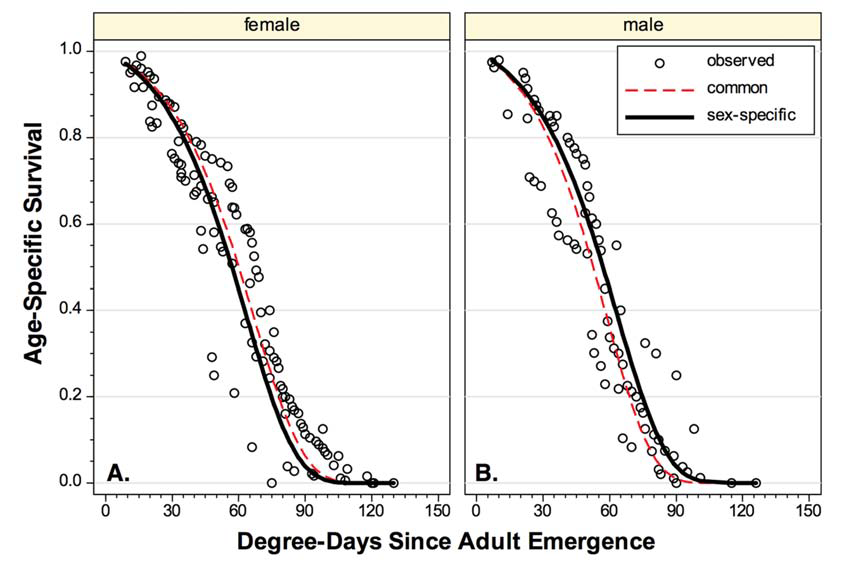
Age-specific survival curves for *Cydia pomonella* with both sex-specific and common Gompertz predictions. A. Females. B. Males.

A comparison of the male and female *l_x_* curves showed that there were only minor differences between them. The *q-q* plot showed that females tended to live an average of 9% longer than males (Figure 2). When a common, non-sex-specific *l_x_* curve was examined, there was < 6 DD difference at both 50 and 90% mortality points between the common curve and the sex-specific curves (Figure 3A, B).

**Figure 4.**
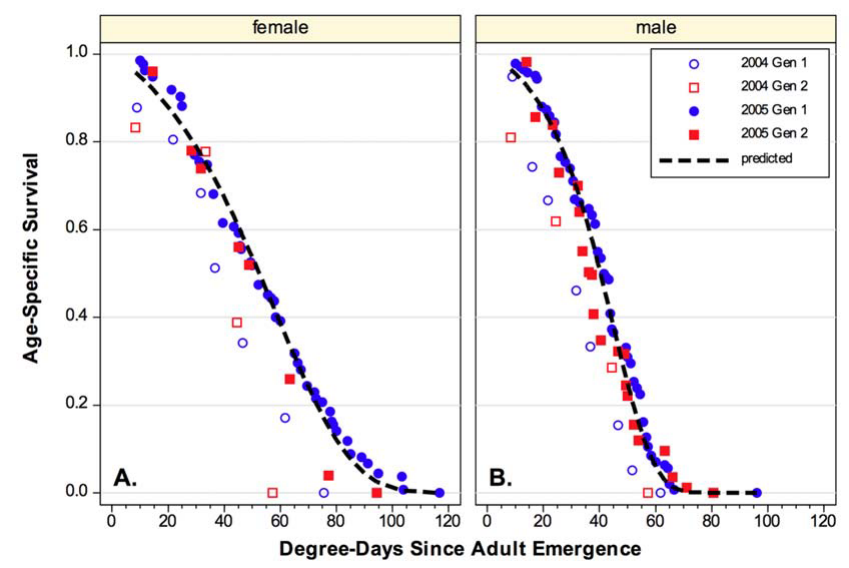
Age-specific survival curves for *Choristoneura rosaceana* on a degree-day basis with sex-specific Gompertz predictions. A. Females. B. Males.

**Figure 5.**
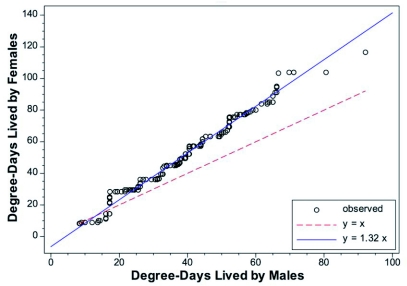
Empirical quantile-quantile plots for male versus female *Choristoneura rosaceana.* If all the data points fell on the *y* = *x* curve, the two distributions would be identical.

The *C*. *rosaceana* data showed that males and female survivorship did not fit a common *l_x_* curve (Figure 4A, B), and sex-specific curves were necessary for good predictability (Table 2). A *q-q* plot showed that on average, females lived 32% longer than males (Figure 5). When sorted by sex, the point of 50% mortality occurred at 38 and 49 DD for males and females, respectively, and at 90% mortality it occurred at 58 and 78 DD, respectively. The maximum longevity was 96 DD and 117 DD for males and females (Table 1), respectively.

## Discussion

These data provide age-specific mortality curves for both *C*. *pomonella* and *C*. *rosaceana* in Washington apple orchards under conditions that reasonably approximate field conditions. While these data are generated using laboratory and not wild moths, studies with codling moths have shown that laboratory selection increases longevity, presumably by both genetic selection and improved nutrition (Cisneros and Barnes 1974). Thus, our data are conservative in the sense that they are unlikely to be underestimating the longevity of moths under natural conditions. A key finding is that because the life spans of both species are relatively short (especially during the summer), a delay in mating of even a few days may reduce population growth simply by reducing the length of the oviposition period. For example, if mating is delayed two days by application of mating disruption techniques and the maximum longevity is eight days, the time available for oviposition is reduced 25%. This factor would operate regardless of other factors associated with delaying mating that might reduce mating propensity and/or fecundity. Although the best possible outcome of mating disruption is the complete suppression of mating, if mating is just delayed a few days rather large reductions in population growth rate can still be achieved.

While simple summary statistics are of less value to understanding population growth, they may still serve as a basis for comparison of our results with older literature that only provides summary statistics. Our results on the maximum longevity of *C*. *pomonella* in the field were similar to that of Geier (1963), who found the maximum longevity of female *C*. *pomonella* caged on apple fruit clusters in the field was 19 d. However, in contrast to Geier, we found a roughly two-fold difference in longevity between the spring and summer generations on a calendar date basis. Working in the laboratory, Hagley (1972) examined *C*. *pomonella* longevity at 15.5 to 30°C and found no difference in longevity by sex. On a DD basis, he found that the average longevity was 270 DD, or roughly twice the maximum longevity that we observed in the field.

In laboratory studies investigating delayed mating using the same colony as used our field tests, we found *C*. *pomonella* females reared at 22°C and ≈ 70% RH and fed honey-water lived an average of 165 DD (13.7 d) and a maximum of 480 DD (40 d) (VPJ and NGW, unpublished observations) or ≈ 3.7 fold longer than in the field. Clearly, the laboratory values far exceed the longevity observed in the field and if used incorrectly could minimize the importance of delayed mating that occurs either naturally (e.g., high winds) or from the use of mating disruption.

A possible criticism of our studies is that we did not control for relative humidity in the experiments. Because the moths could not move from the vials, they could not thermo-regulate (Kührt et al. 2006), nor seek areas of higher RH within the tree. The vials were placed within the traps and those were hung within the apple tree canopy, so they would experience an average value for both temperature and RH found within the canopy and would not be specific to leaves, bark, etc. that they may normally seek out. However, if RH were a key determinant of longevity in our studies, the variation in RH profiles experienced between generations and years would have dramatically increased the variation in the *l_x_* curves that could not be explained by the simple heat-unit based model used. In both species tested, there were no significant differences in the shape of the *l_x_* curves related to generation or year, which strongly suggests that the RH typically found within a Washington apple orchard environment has a relatively minor effect on longevity compared to temperature. Part of this may be a result of the fact that temperature is related statistically to RH in the orchard environment with RH decreasing in general as the temperature increases (over 2003 and 2004 times when the experiments were conducted; %RH = 121.6 - 4.7 × °C + 0.052 × °C^2^, R^2^ = 0.69). In addition, laboratory experiments on adult longevity found that it was unaffected by RH from 45–95%; other humidities were not tested (Hagley 1972).

There is far less laboratory and field data available for *C*. *rosaceana* adult longevity than for *C*. *pomonella.* Sanderson and Jackson (1909) found unfed females had an average longevity of 162 DD (14.6 d), with a maximum longevity of 222 DD (20 d) at a constant 21.1°C. In laboratory studies at 22°C and ≈ 75% RH using the same source colonies of *C*. *rosaceana* as we used in the field tests, we found the average longevity of females provided with honey water solution was 173 DD (14.4 d) and maximum longevity was 408 DD (34 d) (VPJ and NGW, unpublished observations). Thus, similar to the trends found our *C*. *pomonella* comparisons, the maximum *C*. *rosaceana* longevity in the two laboratory studies were between 1.9 and 3.5 fold longer than observed in our field data.

While physiological time is beneficial for predicting the mortality of moths not exposed to predation, there are some quirks associated with its use. In part, this is because the data that was used to determine the longevity predictions were taken daily, and degree-days were thus calculated on a daily basis. However, unlike calendar time, physiological time intervals are not constant, but vary depending on temperatures experienced. This can lead to erroneous conclusions when standard statistical tests are used to compare the DD requirements for percentage mortality of a stage between two categories. For example, a difference of 10–15 DD may be statistically different, especially if the sample size is large. However, 10–15 DD may be less than a day at the temperatures that occur during any given period. Thus, the statistical difference may be of only minor biological relevance. In our data, there were statistical differences between predicted male and female *C*. *pomonella* survival curves, however in terms of practical application, the differences were inconsequential. This is particularly apparent when considering the average degree-day accumulation per day was ≈ 14 DD per day in the summer and ≈6 DD per day in the spring.

Perhaps the largest impact of these data may not be in of improving scientific predictions or models, but in helping to provide the biological insight needed to help mitigate quarantine issues. For example, these data should be useful in negotiating the *C*. *pomonella* quarantine issues with Taiwan (APHIS-PPQ 2005). Our studies show that the maximum longevity of *C*. *pomonella* in the field in the absence of natural enemies and without exposure to pesticides is 21 days or 130 DD (Table 1). Thus, if separate accidental introductions of *C*. *pomonella* occurred ≈21 d apart or longer, there would not be an increased risk of establishment. Therefore, the use of these data helps preserve the market while also continuing to protect the local industries.
